# People, Patches, and Parasites: The Case of Trypanosomiasis in Zimbabwe

**DOI:** 10.1007/s10745-017-9929-y

**Published:** 2017-09-13

**Authors:** Ian Scoones, V. Dzingirai, N. Anderson, E. MacLeod, L. Mangwanya, F. Matawa, A. Murwira, L. Nyakupinda, W. Shereni, S. C. Welburn

**Affiliations:** 1 ESRC STEPS Centre, Institute of Development Studies, University of Sussex, Falmer, Brighton, BN1 9RE UK; 2 Centre for Applied Social Sciences, University of Zimbabwe, PO Box MP167, Harare, Zimbabwe; 3 The Royal (Dick) School of Veterinary Studies and the Roslin Institute, University of Edinburgh, Roslin, EH25 9RG UK; 4 Division of Infection and Pathway Medicine, Edinburgh Medical School, College of Medicine and Veterinary Medicine, The University of Edinburgh, Chancellor’s Building, 49 Little France Crescent, Edinburgh, EH16 4SB UK; 5 Department of Geography and Environmental Science, University of Zimbabwe, P.O. Box MP167, Harare, Zimbabwe; 6 Tsetse Control Division, Ministry of Agriculture, P.O. Box CY52, Causeway, Harare, Zimbabwe

**Keywords:** One Health, Trypanosomiasis, Tsetse fly, Socio-ecology, Zimbabwe

## Abstract

Understanding the socio-ecology of disease requires careful attention to the role of patches within disease landscapes. Such patches, and the interfaces between different socio-epidemiological systems, we argue, have important implications for disease control. We conducted an interdisciplinary study over three years to investigate the spatial dynamics of human and animal trypanosomiasis in the Zambezi valley, Zimbabwe. We used a habitat niche model to identify changes in suitable habitat for tsetse fly vectors over time, and this is related to local villagers’ understandings of where flies are found. Fly trapping and blood DNA analysis of livestock highlighted the patchy distribution of both flies and trypanosome parasites. Through livelihoods analysis we explored who makes use of what areas of the landscape and when, identifying the social groups most at risk. We conclude with a discussion of the practical implications, including the need for an integrated ‘One Health’ approach involving targeted approaches to both vector control and surveillance.

## Introduction

The relationship between ecological and social drivers of disease dynamics is increasingly recognised (Machalaba *et al.* 2015; Bardosh 2016). However, standard disease control approaches tend to adopt a blanket approach, ignoring complex socio-ecologies in fast-changing landscapes (Scoones 2014; Cunningham *et al.* 2017). We argue that more careful attention needs to be paid to spatial dynamics and the role of habitat patches linking disease transmission cycles.

We focus on the case of the parasitic disease trypanosomiasis in the Zambezi valley in Zimbabwe. Despite the intensive control efforts over many decades, the tsetse fly disease vector persists and human and animal trypanosomiasis remains an important economic and health burden. In rapidly-changing ecosystems characterised by increasing habitat fragmentation linked to growing settlement and agriculture, disease vectors are often found only in certain habitat patches. These are sites where people, animals, and pathogens mix, and where transmission cycles intersect. However, standard approaches to both disease surveillance and control do not pick up on such spatial dynamics.

We argue that greater attention needs to be focused on the interactions of people (and so social and cultural factors), patches (and changing habitats and ecologies), and parasites (and the spatial patterns of disease transmission). Patches are biophysical features, marked out by particular topographies, associated with certain vegetation and soil types and animal populations (Pickett and Cadenasso 1995), but they are also part of social landscapes (Scoones 1991). They sometimes carry with them cultural-religious significance, and they are always shaped by an intersection of social relations, institutional dynamics, and political contestation. Disease transmission dynamics also have spatial dimensions that intersect with shifting socio-ecologies. For trypanosomiasis, changes may occur from a ‘sylvatic cycle,’ where wildlife populations are the major hosts for both the parasite and tsetse fly, to a ‘domestic cycle,’ where livestock become the major hosts (van den Bossche and Delespaux 2011; van den Bossche *et al.* 2010). Wildlife hosts may support long-term maintenance of strains that are more pathogenic and which, under changing ecological conditions, may spill over into livestock populations.^1^ When transmission cycles come into contact – for example in patches within changing landscapes – disease dynamics may fundamentally change, with major consequences.

In the Zambezi valley in Zimbabwe, different transmission cycles co-exist. Wildlife persist in high densities within the national parks and safari/hunting areas, some of which have suffered encroachment from settlers, including those ex-farm workers who were evicted following land reform after 2000. Wildlife also migrate up river valleys and gorges into the settled areas during the dry season, encouraged by various wildlife programmes. The transmission cycles are neither separated by time (transforming from one to the other), nor in space (wildlife areas contrasted with settled areas). Instead, they are coincident and overlapping, with human actions resulting in continuous connectivity. Through an examination of the distribution of vector populations, habitat changes, and social and cultural factors, this paper explores why it is that trypanosomiasis persists despite massive and sustained vector control efforts, and what should be done about it.

Following a presentation of our methodology, we first explore where tsetse flies are found in the landscape, and the prevalence of trypanosome parasites in tsetse flies and livestock. We then attempt to explain this pattern through a combination of a habitat niche model, derived from satellite and vector monitoring data, and insights from local communities, who point to the importance of particular patches as vector reservoirs. We conclude that a focus on patch dynamics helps explain why vector-borne diseases, such as trypanosomiasis, persist despite substantial control efforts. Habitat patches, and the interfaces between different socio-epidemiological systems, we argue, have important implications for an integrated ‘One Health’ approach to disease control, and require an integrated, interdisciplinary approach to understanding and action.

## Understanding Disease Dynamics

In a study from 2013 to 2015, we examined the impacts of trypanosomiasis on livestock and humans in Hurungwe district in Zimbabwe. The research involved an interdisciplinary team, including spatial ecologists, geographic information systems specialists, entomologists, parasitologists, veterinarians, social anthropologists, and development policy experts, working with government officials and local villagers.

**Fig. 1:**
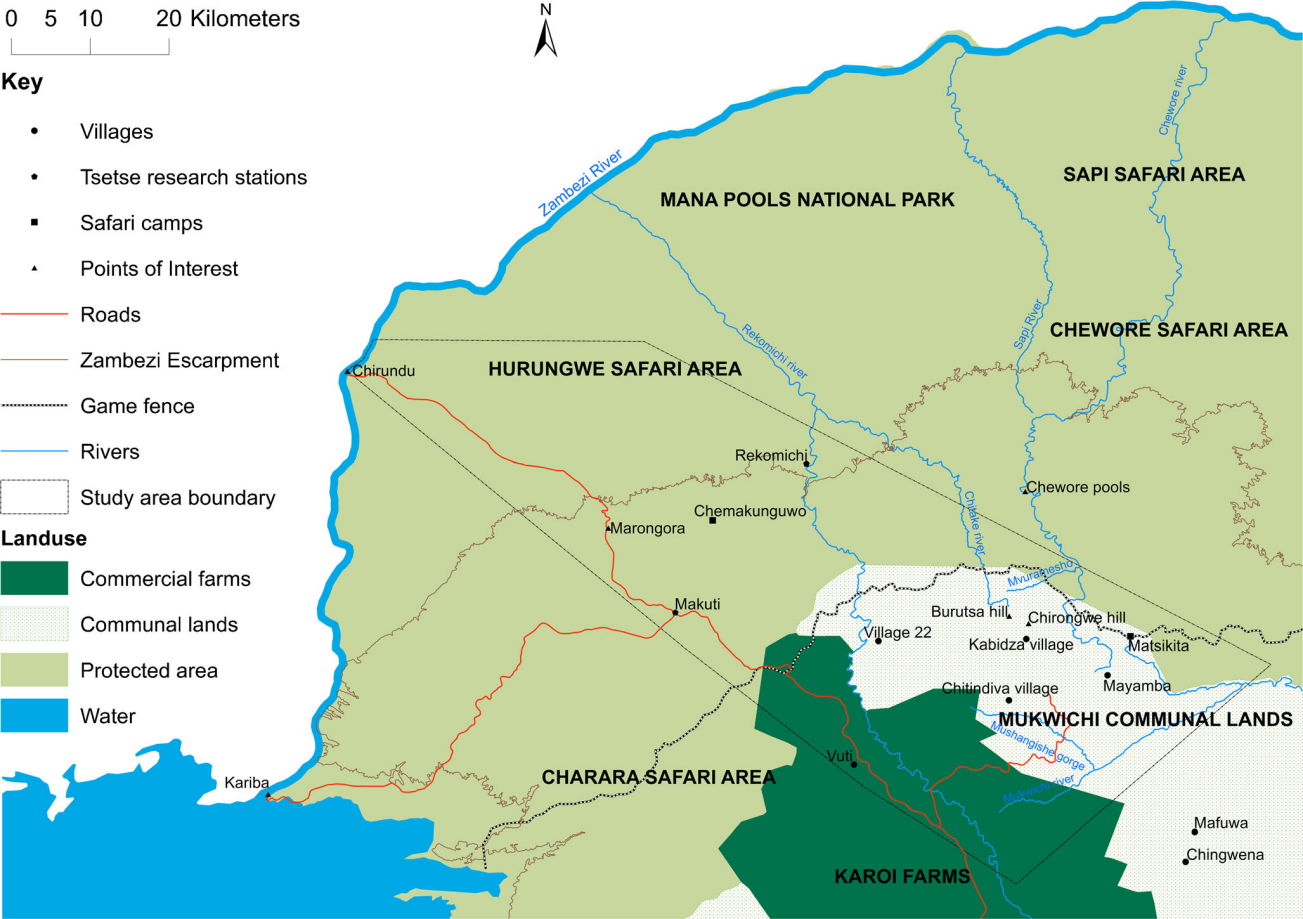
Study area

The study area stretched from the Zambezi valley floor to above the escarpment, encompassing a range of land-uses, including large-scale farms, smallholder farming (communal areas and resettlement areas), safari hunting areas, and national parks (Fig. 1). Different elements of our study took either a wider 110 km × 35 km transect (for satellite image/aerial photo analysis) or a more focused area, centred on Mukwichi communal area (for livelihoods studies and livestock parasitology, and vector distribution analysis), where core, peripheral, and buffer zone villages were contrasted.

Tsetse fly catches were recorded along the 110 km transect on 12 fixed fly rounds, spaced 10 km apart and each 3 km long. Surveys were undertaken twice a month for the duration of the study period. We also analysed cluster trap data from 1996 to 2014 from across the transect. For parasite characterization, DNA extraction was performed on the 213 flies trapped.^2^

A trypanosome survey of livestock populations was conducted in the core and peripheral village area, and blood from 400 cattle and 222 goats was sampled from 19 villages.^3^ Systematic sampling with a random start point was used to select cattle in ten clusters, each linked to a dip tank. The sampling interval was varied between clusters to ensure that equal representation was achieved. The dip tanks covered all villages in the core and peripheral areas and some served more than one village. Goats were sampled using a two-stage cluster sample procedure, using the 19 villages in the study area as the clusters. Within each village cluster, five households were randomly selected and all goats owned by the household sampled.^4^

We used the maximum entropy (Maxent) habitat niche modelling approach (Phillips *et al.* 2006) to predict the spatial distribution of tsetse flies in the study area as a function of elevation, topographic position index, and Landsat satellite derived Normalised Difference Vegetation Index (NDVI).^5^ Additional information on rainfall patterns, temperature, and wildlife presence was collected from the Zimbabwe Met Office and Zimbabwe Parks and Wildlife Management Authority.

Finally, a household survey was undertaken in two areas, around Chitindiva (core) (*N* = 351, in 7 villages) and Kabidza (peripheral) (*N* = 236, in 7 villages), both in Chundu ward in Mukwichi communal area (Fig. 1). Random sampling resulted in 587 out of the 1100 households being interviewed as part of a questionnaire survey. Such a survey was not undertaken in the illegal ‘buffer’ zone settlements due to on-going sensitivities and a lack of a formal listing of households to sample from, as households are dispersed and not in distinct, named village areas. However in all sites – core, peripheral, and buffer - participant observation was combined with key informant interviews and focus group discussions. Archival information from government offices and the National Archives of Zimbabwe was consulted where available. Additionally, participatory mapping exercises were undertaken with two different groups of 15, with varied age/gender representation. Each group was made up of a mixture of villagers from both the peripheral (Kabidza) and core (Chitindiva) areas. The mapping was linked to a scenarios exercise, which discussed, in relation to maps created by villagers, the distribution of tsetse in the past (1980s to 1990s), present (2000s), and future (post-2024), under different demographic and economic conditions.

## Tsetse Flies and Trypanosomiasis in the Zambezi Valley

Trypanosomiasis is a vector-borne, neglected tropical disease that has devastating impacts on human and animal populations across Africa (Hursey 2001). Approximately 10 million km^2^ are infested by tsetse flies in Africa, and economic losses caused by the disease may be as much as US$4.75 billion per year (FAO 2014). Seventy million people are at risk of contracting the human form of the disease, human African trypanosomiasis (HAT), also commonly referred to as sleeping sickness (Simarro *et al.* 2012), resulting in a significant health burden (Fèvre *et al.* 2008).

In Zimbabwe, the acute form of HAT caused by *Trypanosoma brucei rhodesiense* occurs sporadically where the tsetse vector exists alongside human populations. Several species of trypanosome are pathogenic in livestock causing animal African trypanosomiasis (AAT or *nagana*), resulting in severe production losses. In Zimbabwe, two species of tsetse fly (*Glossina pallidipes* and *G. morsitans morsitans*) are present and contribute to the transmission of HAT and AAT. *G. pallidipes* are found predominantly in dense woodland or thicket, whereas *G. morsitans* thrive in more open woodland habitats. Both density and distribution vary seasonally, with fly populations becoming more widely dispersed in the rains and concentrating near water sources in the dry season (Torr *et al.* 2012). Host presence influences fly distribution as tsetse are dependent on blood meals from domestic livestock and wildlife (Anderson *et al.* 2011). Detailed studies of tsetse biting behaviour have shown how precise locations, timings, and the colours and patterns of clothing have important effects on infection rates (Torr and Vale 2015). Herding practices also have an impact, with larger, mixed herds being more attractive to flies (Torr and Mangwiro 2000). Human and livestock exposure to tsetse is therefore affected by a range of factors, including herding, hunting, and farming practices, as well as the movement of wild animal species.

Tsetse flies take refuge in micro-climatic niches, such as warthog burrows, where they deposit their pupae. Temperature and humidity have an important impact on fly distribution, largely through the effect of altitude (Terblanche *et al.* 2008). Tsetse flies exhibit opportunistic feeding behaviour and can survive on blood meals from unusual hosts. Physiologically and behaviourally, tsetse flies are therefore highly adaptable and resilient, making them an ideal vector in a changing environment. However, tsetse flies are also slow breeders: an adult female deposits between only one and eight pupae, and expansion of range is constrained by reproductive biology (Hargrove 1988).

Over the last century, various tsetse control measures have been deployed in Zimbabwe, including bush clearance, extermination of wildlife, ground and aerial spraying with insecticide, bait technologies, and sterile insect release (Scoones 2014). The greatest impact on tsetse populations, however, has arisen from planned and uncontrolled movements of people into tsetse-infested areas. Land clearance for agriculture, combined with the use of crop-spraying chemicals for cotton and tobacco, for example, has resulted in the pushing back of tsetse populations.

**Fig. 2:**
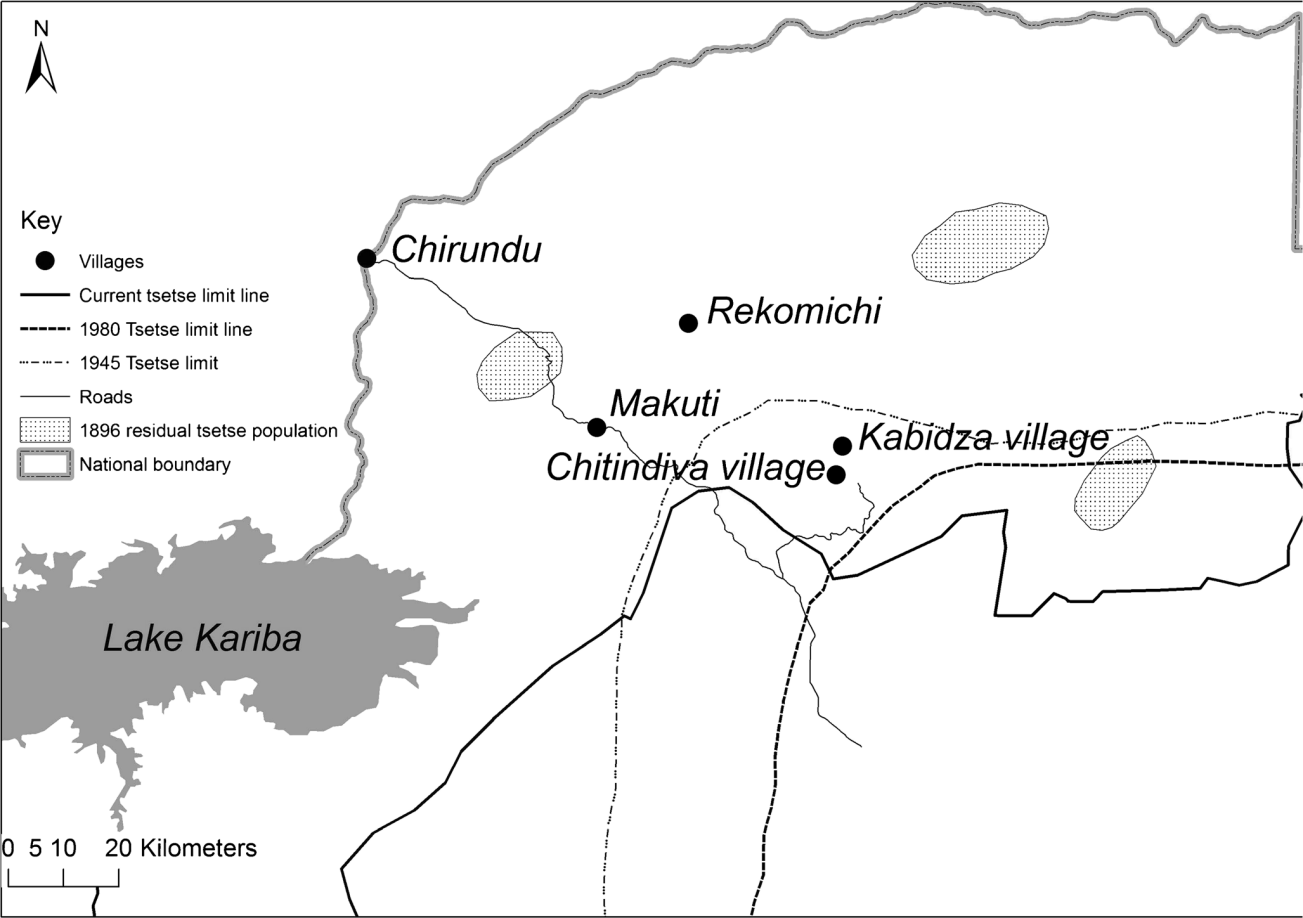
Approximate tsetse limits in 1896 (post-rinderpest), 1945 (post World War II), 1980 (independence), 2016 (current) (extended from Lovemore 1994)

Since the end of the nineteenth century, tsetse fly populations have fluctuated dramatically in the study area. Maps from the early 1900s show populations restricted to a few small areas in the aftermath of the rinderpest pandemic, which eliminated wildlife and livestock populations across Africa between 1896 and 1898 (Jack 1914; Ford 1971). In our study area, remnant focal areas were found near the Zambezi River (Lovemore 1994; Fig. 2). As animal populations recovered from rinderpest, the area inhabited by tsetse expanded. But with the colonisation of what became Southern Rhodesia and the establishment of a settler economy, tsetse control became a priority as white-owned commercial farms were established (Matzke 1983).

During the liberation war for Independence, control efforts ceased in many areas, and the Zambezi valley was an important focus for the guerrilla struggle (Lan 1985). Tsetse populations expanded again and, following Independence in 1980, the new government, together with international donors, made tsetse clearance for settlement, agricultural development, and tourism in the valley a m**a**jor economic and development priority. For example, the European-funded Regional Tsetse and Trypanosomiasis Control Programme, which ran from 1986 to 2000, involved a massive effort to ‘push back’ the tsetse ‘belt’ (Scoones 2014) (Fig.2).

After 1980, policies encouraged inwards migration into the Zambezi valley, and many settlers moved spontaneously from other parts of the country, initially growing maize, but later cotton and more recently tobacco (Chimhowu and Hulme 2006). In Mukwichi communal area, for example, the total human and livestock populations increased from 9,758 in 1992 to 15,388 in 2012 (ZIMSTAT 2013).

## The Spatial Ecology of Tsetse and Trypanosomiasis in Hurungwe, Zimbabwe

**Fig. 3:**
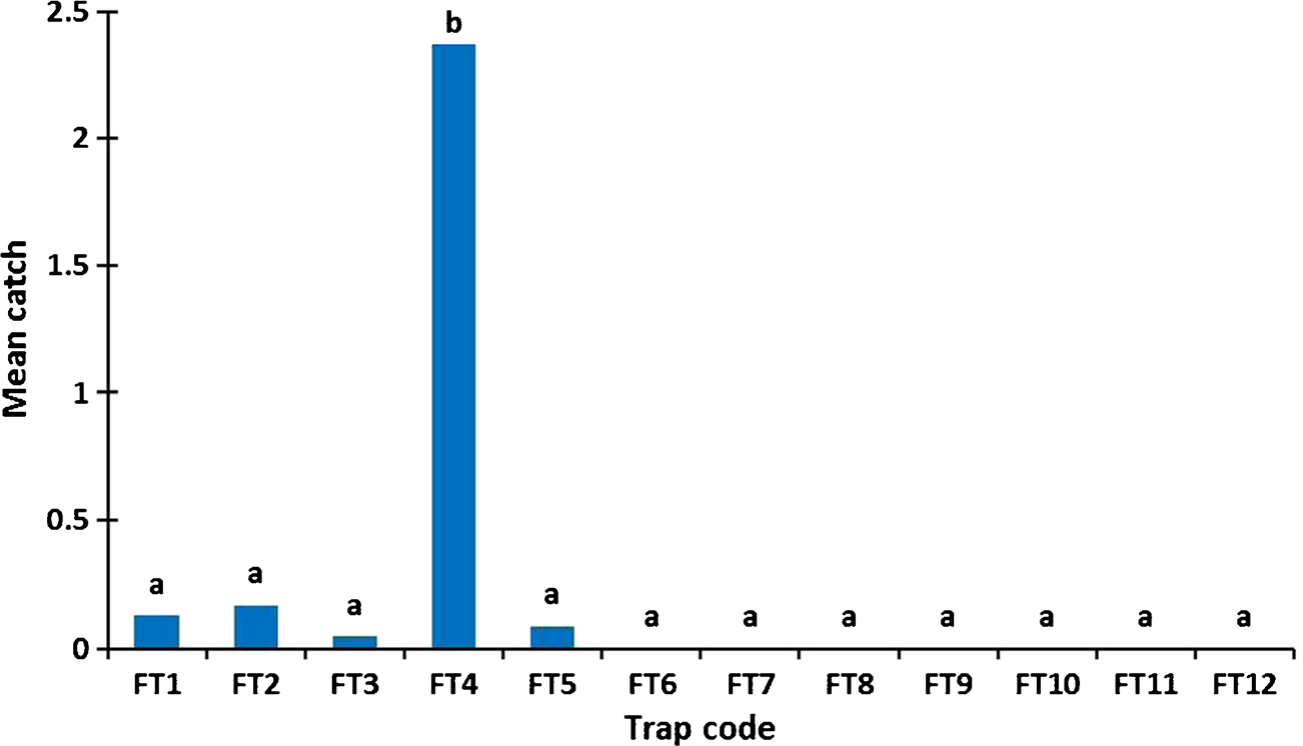
Mean tsetse fly catch per round along a 110 km transect from north (FT1) at the valley floor to south (FT12) above the valley floor, between March and November 2014

Our data showed a declining tsetse population density gradient both north and south from the Zambezi escarpment. The highest catches were observed in fly round 4, located immediately above the escarpment, with most flies caught using mobile baits being *G. m. mortisans* rather than *G. pallidipes* (Fig. 3). Cluster trap data from across the transect show declining tsetse populations from 1996 to 2014. In the settled and cultivated Mukwichi area, no flies were caught in cluster traps or fly round transects since 2007.

Almost half of the sampled tsetse flies (48.8%) were found to contain trypanosome DNA. The most prevalent species of DNA identified was *T. vivax* (in 32% of flies), followed by *T. brucei*, then *T. godfreyi*, *T. congolense*, *T. simiae*, and *T. simiae Tsavo*. This pattern was consistent across the two fly species (*G. m. mortisans* and *G. pallidipes*) and fly sex.

**Fig. 4:**
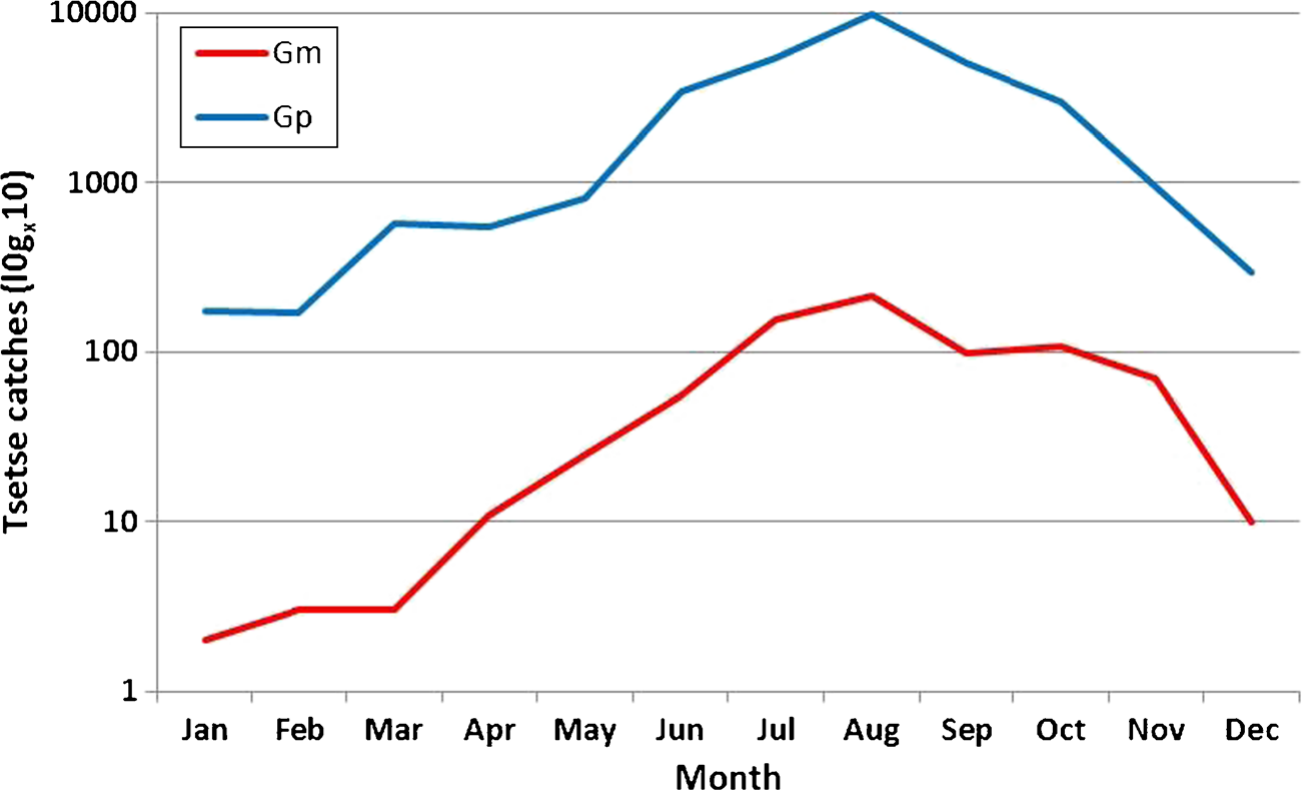
Tsetse fly incidence across seasons (by species – Gm = *G. m. mortisan*s; Gp = *G. pallidipes*) at Rekomichi research station, 2012

In those areas where tsetse flies persist, their presence is seasonal. Data collected during 2012 from traps at Rekomichi research station in the valley area, show peaks in tsetse populations during the dry season in August. Catches were lowest during the wet season (November to March) (Fig. 4). The majority of tsetse flies become infected at their first feed when they are most susceptible (Welburn and Maudlin 1991). Our analysis of mouth parts showed infection rates range from 5 to 10%, and, as would be expected, are significantly higher in susceptible male *G.morsitans* and *G.pallidipes.*

Triangulating the tsetse catch data with local key informant interviews (see below) indicated that transect and cluster trap sampling was revealing only a partial picture. Linear samples across wide areas are less appropriate within a fragmented habitat. Cluster traps in settled areas, often set near dip tanks full of insecticide, also failed to take account of particular patches of suitable vector habitat. As a result we designed a focused patch sampling approach, using sites identified by local people from their participatory natural resource maps. This approach led to trap catches in sites that were close to settled and farmed areas, and confirmed the presence of tsetse in areas for which the long-term sampling programme had not recorded fly presence in over a decade.

**Fig. 5:**
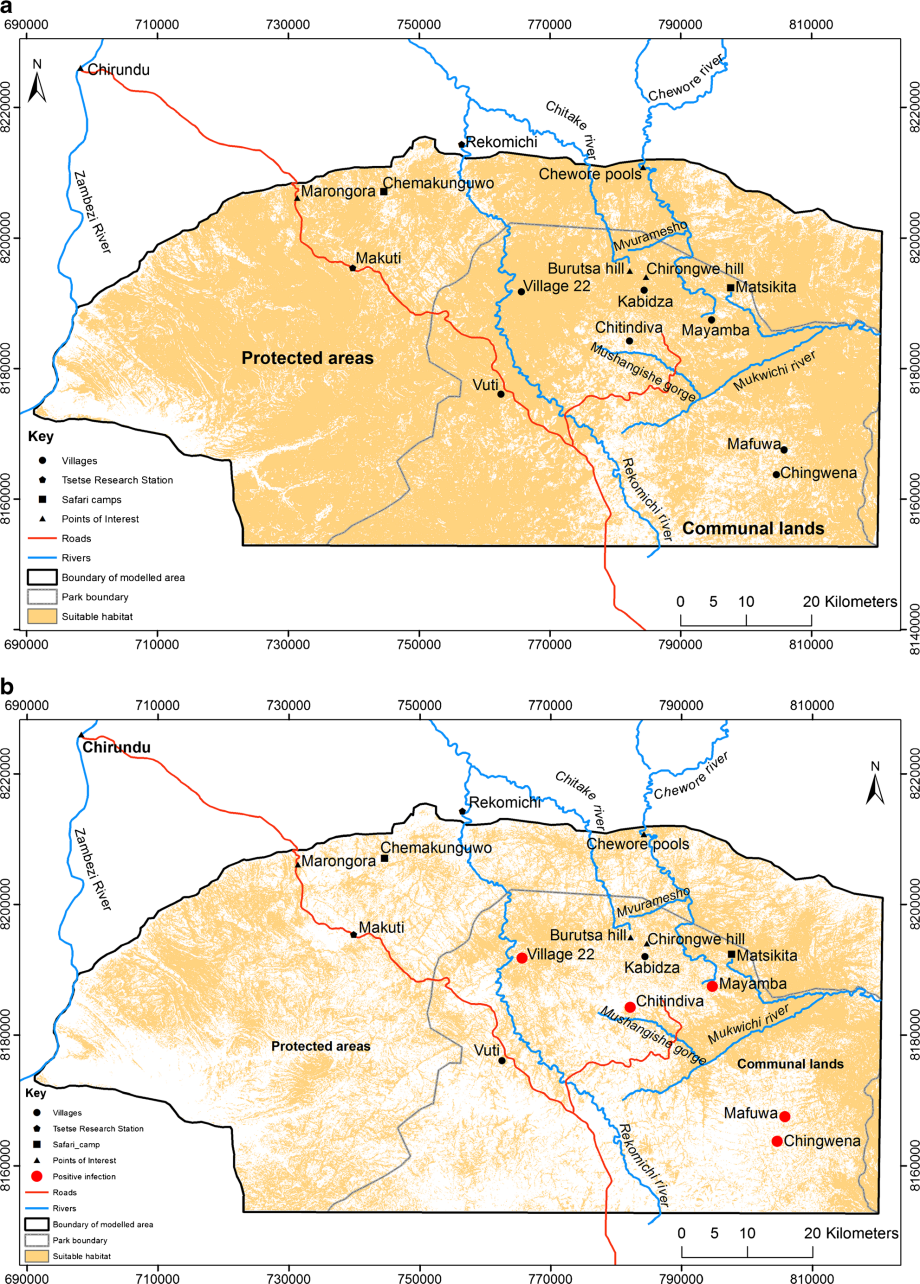
**a** and **b**: Habitat suitability for tsetse flies (combining land cover, elevation and topographic position index) comparing 5a: 1986 and 5b 2008 (also showing site of positive infections of trypanosomes in livestock)

Relating our understanding of vector distribution to presence of disease organisms required the sampling of livestock for presence of trypanosomes. This took place in the core and peripheral farming area, but excluded illegal settlements in the buffer zone. Livestock in villages across these areas were sampled randomly. In four of 19 villages sampled, AAT was identified in cattle. AAT prevalence in cattle indicated clusters of infection, each linked to a particular ‘patch’ where tsetse presence was most likely (Fig. 5b). In each case they are found in sites where tsetse fly habitat patches are nearby. These were all near areas where local people had identified the persistent presence of tsetse flies and where our focused cluster trap sampling had found tsetse flies.

Thus, positive samples were discovered in Chitindiva village, where 7.5% of cattle were infected (*N* = 40, mix of *T. brucei* and *T. vivax*). Cattle are moved from here to the Mushangishe gorge nearby for grazing, where dense bush exists and tsetse flies thrive. The gorge is also a migration route for wildlife in search of water, particularly in the dry season. In Mayamba, 1.25% of cattle (*N* = 80) were infected with *T. vivax*. This is a village close to the game fence and, while not as exposed as the illegal settlements in the buffer zone bordering the national park, their animals graze extensively in this area. Here, herded cattle share the same habitat as wildlife, including a number of species that are intermediate hosts of trypanosomiasis. At Village 22, 5% (*N* = 40, mix of *T. brucei* and *T. vivax*) of cattle were infected. Village 22 is at the edge of the forested Hurungwe Safari Hunting Area, where there has been a recent upsurge of infected tsetse, and where HAT cases have occurred. This area is again has dense vegetation, a suitable habitat for tsetse flies, and is inhabited by a variety of wildlife. In Chingwena, 10% of cattle were infected with *T. brucei* (*n* = 40), while in Mafuwa, there was one goat identified with a dual infection. These villages are near the buffer zone, as well as riverine forest patches - all ideal habitats for the tsetse fly vector.

The 15 villages where no trypanosomes were found were largely in the core area, where vegetation had been cleared, or in the peripheral zones further away from streams or rivers, such as the two villages at Kabidza (Fig. 5b). No *T. b. rhodesiense* were identified in any sample, suggesting the risk is principally AAT, rather than HAT. Villagers indicated that cattle were often treated with the prophylactic trypanocide Isometamidium chloride three to four times per year, and this will have reduced the prevalence of trypanosomes across all villages, making detection more difficult.

Data on HAT mortalities are poor in Zimbabwe. Only eighteen cases were reported between 2012 and 2015, with a peak in 2012 when 11 cases were reported. Long-term monitoring of human trypanosomiasis in Zimbabwe shows very low incidence (Katsidzira and Fana 2010), so these new cases represent an unusual shift. HAT is significantly under-reported, as symptoms are similar to malaria and other fevers (Odiit *et al.* 2005). Most recorded cases are tourists, hunters, or those working with the national parks service. For example, the cases in 2012 included two tourists and a professional hunter, while a game ranger succumbed to the disease in 2010. We were unable to undertake human blood sampling during our study, but despite not finding *T. b. rhodesiense* in the livestock blood sampling, we expect deaths due to HAT to be higher than those recorded.

## Spatial Patterns and Changing Disease Ecologies

Landscape and habitat patterns have had a major impact on how disease dynamics operate in a relatively high population density setting with fragmented habitats. There is no longer a tsetse ‘belt’ as depicted in the conventional maps or a ‘front’ across which to fight the fly, but instead a series of discrete but interconnected patches dispersed across landscapes. These are rapidly changing as new people arrive in the valley, and agricultural and livestock keeping practices change. Communities are fighting, as they put it, a ‘guerrilla struggle’ against tsetse flies, with occasional ‘skirmishes and contacts’, rather than a more conventional ‘war’ with a visible ‘front,’ detectable through standard sampling techniques. Understanding this new socio-ecology of trypanosomiasis has major implications for disease control, and requires adaptation of monitoring and control methods.

The challenge that infected tsetse flies pose is directly related to tsetse abundance, which can be inferred from modelling tsetse distribution using remotely-sensed imagery. Overall, suitable tsetse habitat decreased between 1986 and 2008 (Figs 5a and b). This pattern was driven by a combination of land-use change, settlement patterns, and rainfall levels. We found that riverine and escarpment habitats showed less change over time, being less affected by land-use change or settlement, and so had a higher proportion of suitable habitat.

The expansion of agriculture results in the fragmentation of wildlife habitat, reducing wildlife populations (Baudron *et al.* 2010; Matawa *et al.* 2012). In our study area, wildlife inhabit the buffer zone and hunting area, and as part of dry season migrations along the Mushangishe gorge. Satellite image analysis confirms these areas are those where the most suitable tsetse habitat persists. These areas thus represent important disease and vector reservoir patches and interfaces between the sylvatic and domestic transmission cycles, and indeed were where AAT was observed in livestock.

On participatory maps, our local informants identified patches where tsetse abundance was notably higher. These included the wooded escarpment; the Mushangishe gorge near Chitindiva village, which drains into the Chewore river, a major tributary of the Zambezi; the Chitake river forest area; the buffer zone bordering the national park and the Hurungwe Safari Hunting Area; plus some additional small sites within the farming areas in wooded valley bottoms, around pools and on hills.

These patches may become more significant at certain time periods; for example during long dry spells with high temperatures when fly populations will be concentrated and exposure to AAT/HAT consequently higher. Records for our study site show high temperatures during drought periods from September to early December in several years recently. This may be related to an increase in reports of trypanosomiasis following these episodes. In 2016, the major El Niño event caused a similar pattern and we can expect further shifts in tsetse fly populations due to climate change, as flies retreat into cooler, wetter patches in the landscape.

Combining the data on tsetse fly presence and environmental/land-use patterns at an appropriate spatial scale, strong correlations are observed with tsetse fly infestation levels, habitat suitability, and wildlife presence. But in order to understand disease risk, we need to know more about how people and animals interact with patches at these crucial interfaces of disease transmission.

## The Socio-Ecology of Disease Transmission

To probe our understandings of infection dynamics, we complemented our studies of entomology, parasitology, and spatial ecology with analysis of livelihoods and social relations in several sites within the study area in Mukwichi communal area, asking how people construct livelihoods in this changing landscape and interact with tsetse and trypanosomiasis.

This work throws important light on the socio-ecology of patches – how they are constructed, perceived, and become enmeshed with social and political relations. Populations in this area have been subject to a series of displacements and inward migrations over time. A key distinction is between the core village area around Chitindiva, the peripheral villages near the game fence, and the illegal settlements in the ‘buffer zone.’ The core villages have been settled for over 60 years, when people were moved to this area from the valley floor following the establishment of protected areas, the damming of the Zambezi River, and the creation of Lake Kariba. The peripheral villages were established through inwards migration, particularly from the 1980s, while the buffer zone settlements have sprung up, especially since the land reform of 2000.

The original Korekore residents compete with Karanga migrants for control of the natural resources on this area. These migrants, known locally as ‘*vauyi,*’ often have good connections to urban businesses and political elites, and have a strong economic base, built initially from cotton farming, and more recently tobacco farming. Since the early 1990s, over 14,000 cattle have moved into the area, grazing in the village areas. More recent migrants are from diverse origins, including from outside Zimbabwe, often former farm and mine workers displaced following land reform and the economic crisis of the 2000s. These migrants joined others in the buffer areas, carving out farming areas in the bush known locally as ‘*mapeto.*’ They live within the wildlife areas, cultivating, hunting, and some keeping cattle.

Patches in this highly fragmented and now relatively heavily populated landscape are maintained in part by physical attributes and topography, such as the inaccessible gorge and river valleys, and are also sustained by political, institutional, and cultural processes. Hunting and buffer areas are subject to competing claims among the national parks authorities, hunting concessions, carbon forestry companies, the rural district council, and local chiefs and headmen, all of whom claim rights over the land and resources. Until recently the net effect has been only limited settlement in such areas, and as a consequence a persistence of suitable tsetse fly habitat.

Local religious and cultural beliefs also play a role in maintaining patches. The Chirongwe and Burutsa hills are sacred sites and ancestral burial grounds and the spirits of founding lineages are said to inhabit the Chewore pools. The forested areas in such sites are protected by *mhondoro* territorial spirits, and are overseen by powerful spirit mediums who often also live in the forests, offering healing remedies to local people. Any person settling in these areas and establishing fields invites a curse from the spirits and punishment from traditional authorities. A person could be fined an ox depending on the nature of infringement. The newer migrants ignore the traditional authorities; being largely Christian they do not follow the Korekore traditions. Four migrants are currently mechanically pumping water from the sacred pools to water tobacco nurseries, to the displeasure of chiefs and the original residents.

The core, peripheral, and illegal ‘buffer zone’ villages are quite different. Land holdings and areas under cultivation are greater in the core villages than either peripheral or buffer zone sites, with an average of 6.6 ha in total, of which 4.7 ha are cultivated. Livestock ownership is similar between the core and peripheral villages, with slightly higher numbers on average in the peripheral villages, where grazing is more abundant (4.1 versus 4.2 cattle per household and 6.4 versus 6.7 goats per household), and lowest in the buffer areas. Overall, asset levels (including farm equipment such as tractors, car ownership, etc.), inputs into agriculture (fertilisers, pesticides, etc.), labour hiring levels, and home quality (brick and tin roof rather than mud and thatch) are higher in the core villages and lowest in the buffer zone areas. A community extension worker echoed our survey findings:

Hurungwe is a divided world. In the core villages you find ‘*shoroma:*’ people who have some means, people with many wives, people with cattle. But if you go to the other areas, all you see are ‘*vanashuro*:’ people cultivating with hoes, without cattle or even goats (Interview Chitindiva, 15 November 2013)

There are within-site variations also. Survey analysis revealed that most of the land, cattle, and goats in both the core and peripheral villages are owned by a few individuals. The distribution of land ownership in the core villages ranges from 2 to 14 ha. While the local elite in the core villages (‘*shoroma’* ) own cattle and goats, and suffer the burden of AAT, their household members do not necessarily do the herding, and so avoid exposure to tseste and risk from HAT. Herding labour is usually hired from poorer households, often from the peripheral and buffer zone villages, and herders may come and live in the livestock owners’ homes for seasonal herding.

Gender and age are significant factors influencing livelihood occupations and this has a major influence on where people go – including to our identified patches – and their exposure to HAT and other diseases. In all villages, but particularly the buffer zone villages where no formal welfare support by the state exists, women, particularly poorer women, are involved in gathering fruits, caterpillars, insects, and honey, including in the wildlife areas, mountains, and down into valleys and gorges. Korekore men, often associated with particular *midzimu* (ancestral spirits) and *mhondoro* (territorial spirits), are the majority of hunters, and learn this skill often across generations, with spirit associations and hunting practices located in particular families known as *mhare*. These men move in similar areas to women involved in gathering, although sometimes they go beyond ‘*mapeto*’ into protected lands. Herding is undertaken by a mix of hired and family labour (see above), but usually by young men or children. They move with herds in the cropping season (due to cultivated fields) or, in the dry season, collect free ranging herds at the end of the day. Grazing sites include the river valleys, mountains, and gorges, which are away from the cultivated fields, and in the dry season constitute the last ‘key resource’ grazing (Scoones 1995).

Thus rich and poor, young and old, men and women, recent and older migrants and autochthones are all exposed to tsetse flies and the indirect impacts of AAT and direct impacts of HAT in different ways, depending on their occupations and locations. Movement to patches – for grazing, gathering, and hunting in particular – entails a high risk of exposure and residing in the peripheral villages and in the buffer zone is especially risky. Not all individuals or everyone’s livestock is exposed, and social-political-ethnic-cultural relations, as well as patterns of social differentiation, migration histories, and livelihood occupations are important in defining the socio-ecological relations that result in disease incidence and spread (Dzingirai *et al.* 2017a, b).

## Joint Analysis for Sustainability and Health

Our approach was to combine different methods and insights and iterate between them. This occurred between the tsetse fly and trypanosome blood analysis and the GIS spatial habitat assessments, and also between these data and survey information and local discussions with village participants. A series of village meetings was organised, attended by men and women, young and old, short-term and long-term residents. This provided a basis to examine our emerging hypotheses, triangulating with local knowledge and insights.

**Fig. 6:**
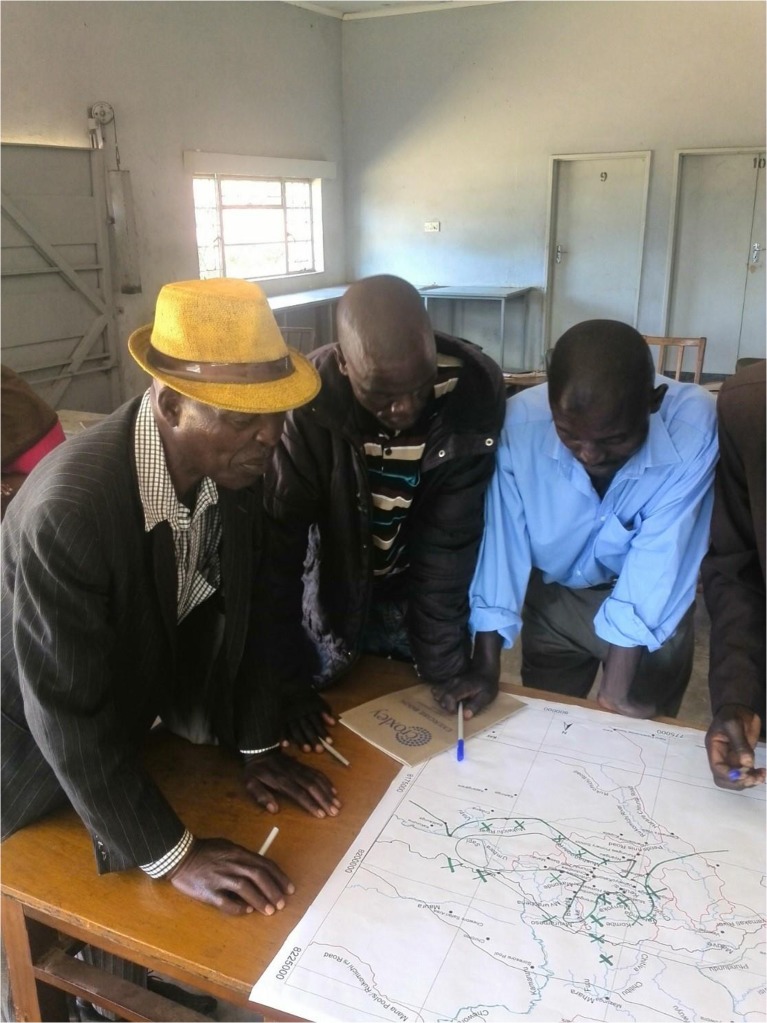
Construction of local maps by a men’s group

This approach prompted a new tsetse fly sampling strategy, and participatory mapping provided the basis for exploring the idea of ‘patches’ and ‘exposure.’ Towards the end of the study we shared the layered GIS maps of past and present land-use and habitat patterns in a workshop, and asked community members to draw their own maps of past, present, and future landscapes, and to discuss the implications for how people interact with tsetse flies (Fig. 6).

All groups emphasised three points. Firstly, that they expected to be continuing to ‘live with the tsetse fly’ (*kugara netsetse*) because wildlife will remain in the area, especially if encouraged by the CAMPFIRE (Communal Areas Management Programme for Indigenous Resources) programme, which allows for the benefits of sustainable utilisation of wildlife through hunting to be shared with local communities and local government. Secondly, that there would be further fragmentation of habitats (*kudimbuka dimbuka*) as people continued to settle and farm. Finally, that migration (‘*kuuya kwevanhu*’) would persist due to the profitability of tobacco and the relative availability of land.

While the presence of wildlife in settled areas would ultimately decline, they suggested that exposure to AAT and HAT would continue within certain patches, less on the ‘frontier,’ as this would be pushed back. As Mr. Chisauka, a village head, observed:

The risks will continue; there cannot be a time we will be completely free because the tsetse fly will be in the Mushangishe gorge, where forests and wildlife can still be found. The tsetse will continue to be found on the banks of Chewore River where we have gardens and where our cattle graze

Local community members informed and corroborated our findings. They believed that, despite continued population growth and vegetation clearance for settlement and farming, including in the ‘buffer zone’ areas, the interfaces between the sylvatic and domestic cycles would persist, with contacts focused on particular patches in an increasingly fragmented landscape. Our findings therefore have important implications for future research design, vector and disease surveillance, and policy priorities.

## Conclusion

An interdisciplinary approach was applied to exploring the socio-ecological dynamics of tsetse and trypanosomiasis in Hurungwe district, Zimbabwe. This highlighted the importance of habitat patches that act as reservoirs for tsetse fly vectors and so sites of transmission. These patches exist at the interface between different transmission cycles, connecting flies, wildlife, livestock, and humans. Understanding how patches are created and maintained and how they are used requires an understanding of socio-ecology, with an historical perspective. An interdisciplinary approach is essential. This must combine both natural and social science investigation, but crucially must involve local participation: in our case, it was only when villagers pointed to particular habitat patches that we were able to interpret our data on tsetse fly distribution, trypanosome presence, and habitat change.

Patches within increasingly fragmented landscapes are created through human actions, and the interfaces between transmission cycles are mediated by livelihood practices, differentiated by gender, age, and occupation. Processes of in-migration into the Zambezi valley have changed land-use, as well as institutions, cultural norms, and authority structures that govern landscapes, and who lives and farms where. Neither the extents nor boundaries of patches are fixed, but are continuously negotiated in social, political, and cultural spaces.

This has major Implications for approaches to disease control. In the past ‘area-wide’ approaches have dominated, involving mass insecticide spraying, vegetation clearance, or wildlife elimination (Scoones 2014). Trypanosomiasis has persisted, even in highly settled areas, bringing disease risks, especially for certain social groups. There is a need to go beyond the idea of managing a tsetse ‘front’ or ‘belt’ to focus interventions in particular sites, informed by spatial analysis and participatory approaches. Mobile bait and trap technologies, implemented and managed by local communities, combined with effective animal health provision, would, we argue, provide a sustainable and highly cost-effective control solution (Torr *et al.* 2007).

Our work also has implications for how vector surveillance is undertaken. Coarse-resolution satellite images will not pick up the highly variegated pattern of fragmented landscapes. Equally, linear transects across large areas or random cluster trapping reveal little in areas where flies reside only in small patches. Application of fine-resolution spatial techniques, combined with purposive sampling, informed through community-led participatory mapping and analysis, are needed. Such approaches can draw on insights from local experience and knowledge, and so help focus control interventions and surveillance efforts more effectively.

Taking a wider, more integrated ‘One Health’ perspective (Zinsstag *et al.* 2015; Bardosh 2016; Cunningham *et al.* 2017) on vector and disease control means focusing on landscapes, livelihoods, and disease as part of complex and dynamic socio-ecological systems. In such systems there will be trade-offs between different land uses. In our case study area, for example, crop farming, livestock keeping, hunting (both informal and commercial), biodiversity conservation, ecotourism, and carbon sequestration projects all competed and in turn influenced disease dynamics in different areas.

We have shown that taking an interdisciplinary and multi-scale approach, combining knowledge from different sources, challenges understandings of disease transmission and so control and surveillance responses. Such a socio-ecological approach, which looks at people, patches, and parasites together, and is rooted in participatory field engagement suggests a more effective way forward for responding to disease threats in complex and changing environments.

## Footnotes

1 As in Uganda, livestock can sustain human trypanosomiasis, which presents as a mild infection in domestic animals but poses a significant risk to humans (Welburn *et al.* 2006).

2 DNA extraction was carried out using the DNAeasy kit. Three sets of PCR were run on each sample using *Wigglesworthia* (FliC), Sodalis (SODGroEL) and trypanosomes.

3 Cattle sampling occurred in cattle from the following villages (or clusters of villages): Burosa (*N* = 40), Chingwena (*N* = 40), Chikore (*N* = 80), Chipapa (*N* = 40), Chitindiva (*N* = 40), Dete (*N* = 40), Mayamba (*N* = 80) and Village 22 (*N* = 40). Goats were sampled in the following villages (or clusters of villages): Chipapa (*N* = 64), Kabidza (*N* = 44), Mafuwa (*N* = 74), Mayamba (*N* = 40). Sites where trypanosomes were discovered are marked on Fig.5b.

4 Blood samples for both species were collected from an ear vein and placed on Whatman FTA cards using a heparinised capillary tube. DNA was extracted from FTA cards using standard protocols and all samples subjected to molecular analysis, enabling the identification of pathogenic trypanosomes.

5 For the modelling process, tsetse occurrence data (*N* = 50) for 1998 were randomly partitioned into a 70% training sample and a 30% test sample (Matawa *et al.* 2015, 2016). The Maxent model determined using the 1998 data was then used to predict tsetse habitat suitability onto the 1986, 1991, 1993, 2002, 2007, and 2008 images. We downloaded cloud-free (less than 10% cloud) 30 m spatial resolution Landsat TM and ETM satellite data in order to estimate vegetation greenness and the 30 m Advanced Spaceborne Thermal Emission and Reflection Radiometer (ASTER) digital elevation model (DEM) to derive elevation in metres and topographic position index (TPI).
